# Esophageal cancer risk is influenced by genetically determined blood metabolites

**DOI:** 10.1097/MD.0000000000040122

**Published:** 2024-10-25

**Authors:** Jieyin Deng, Silin Wu, Ye Huang, Yi Deng, Ke Yu

**Affiliations:** a Department of General Medical Practice, General Hospital of PLA Western Theater Command, Chengdu, China; b School of Clinical Medicine, North Sichuan Medical College, Sichuan, China; c Department of Nursing, Nursing School, Chengdu Medical College, Chengdu, China.

**Keywords:** causality, esophageal cancer, fatty acyl, Mendelian randomization, metabolites, sphingosine

## Abstract

It remains unclear what causes esophageal cancer (EC), but blood metabolites have been connected to it. Our study performed a Mendelian randomization (MR) analysis to assess the causality from genetically proxied 1400 blood metabolites to EC level. A two-sample MR analysis was employed to evaluate the causal relationship between 1400 blood metabolites and EC. Initially, the EC genome-wide association study (GWAS) data (from Jiang L et al) were examined, leading to the identification of certain metabolites. Subsequently, another set of EC GWAS data from FINNGEN was utilized to validate the findings. Causality was primarily determined through inverse variance weighting, with additional support from the MR-Egger, weighted median, and MR-PRESSO models. Heterogeneity was assessed using the MR Cochran *Q* test. The MR-Egger intercept and MR-PRESSO global methods were employed to detect multicollinearity. In this study, Bonferroni corrected *P* value was used for significance threshold. We found 2 metabolites with overlaps, which are lipids. Docosatrienoate (22:3n3) was found to be causally associated with a decreased risk of EC, as evidenced by the EC GWAS data (from Jiang et al) (odds ratio [OR] = 0.620, 95% confidence interval [CI] = 0.390–0.986, *P* = .044) and the EC GWAS data (from FINNGEN) (OR = 0.77, 95% CI = 0.6–0.99, *P* = .042), these results were consistent across both data sets. Another overlapping metabolite, glycosyl-N-(2-hydroxyneuramoyl)-sphingosine, was associated with the risk of ES, with EC GWAS data (from Jiang L et al) (OR = 1.536, 95% CI = 1.000–2.360, *P* = .049), while EC GWAS data (from FINNGEN) (OR = 0.733, 95% CI = 0.574–0.937, *P* = .013), the 2 data had opposite conclusions. The findings of this study indicate a potential association between lipid metabolites (Docosatrienoate (22:3n3) and glycosyl-N-(2-hydroxynervonoyl)-sphingosine (d18:1/24:1 (2OH))) and the risk of esophageal carcinogenesis.

## 1. Introduction

Esophageal cancer ranks as the 8th most prevalent form of cancer globally and stands as the 6th primary contributor to cancer-related mortality,^[1]^ it is globally recognized as the primary contributor to cancer-related deaths,^[2]^ and is associated with an unfavorable prognosis.

In recent times, metabolomics has emerged as a novel platform for the identification of biomarkers, serving as an essential component of systems biology and offering a fresh perspective on investigating the underlying mechanisms of diseases.^[3,4]^ Metabolic science can deepen the understanding of the biological mechanisms of disease by identifying metabolites or modified metabolic pathways.^[5,6]^ Shen and colleagues^[7]^ reported the discovery of 20 metabolites in fresh tumor tissue and mucous membranes of normal tubes using gas chromogram/mass spectrometry. Metabolic techniques have been used to detect, divide and diagnose metabolites related to the progress of the esophageal cancer (EC).^[8,9]^ For example, glucose, fatty acids, glutamic acid, glycine, citric acid, and taurine have been shown to be closely associated with disturbances in the metabolic pathway in the EC.^[9–11]^ Xiaoli Zhang et al^[12]^ found significant changes in lipid metabolism, amino acids, glycolysis, ketones, triglyceride cycle, and energy metabolism in EC patients. Metabolic reprogramming plays an important role in the occurrence and progression of cancer. Zhang, X et al,^[12]^ observed clear metabolic differences between EC patients in relation to lipids, glucose, energy, amino acids, and significant disturbances in the tricarboxylic acid cycle. Jing Xu et al^[9]^ found that the levels of 3 (octanoylcarnitine, hemolyzed opc (16:1), and decanoylcarnitine) were closely related to the therapeutic effect. Analysis of metabolic properties in nonheterogeneous tumors helps to understand metabolism in tumors.^[13]^ Metabolic reprogramming represents cancer-related metabolic changes during tumors, and is a new sign of cancer.^[14]^ Special metabolites can stimulate improper cell division, and some cancer cells depend on certain metabolic pathways.^[15]^ However, there is still a lack of evidence on the causal relationship between metabolites to prevent EC tumors.^[4]^

More recently, Mendelian randomization (MR) has been widely used in studies of the causes of disease. In the absence of randomized controlled trials, MR was the most compelling strategy for exploring the causal relationship between interest and outcomes. MR assesses the causal effects of genetic factor exposure on outcomes by selecting the diversity of individual exposure-related nuclide forms as the instrument variable.^[16]^

This study aims to identify different metabolites and metabolic pathways for progress in EC, predict tumors through metabolic studies, identify unusual metabolic pathways, and provide information on EC progress. Through the examination of variable metabolites, further randomized analysis was carried out with Mendell double samples^[17]^ to illustrate important biological processes to study the relationship between blood metabolites and EC.

## 2. Methods and materials

### 2.1. Study design

**Figure 1.**
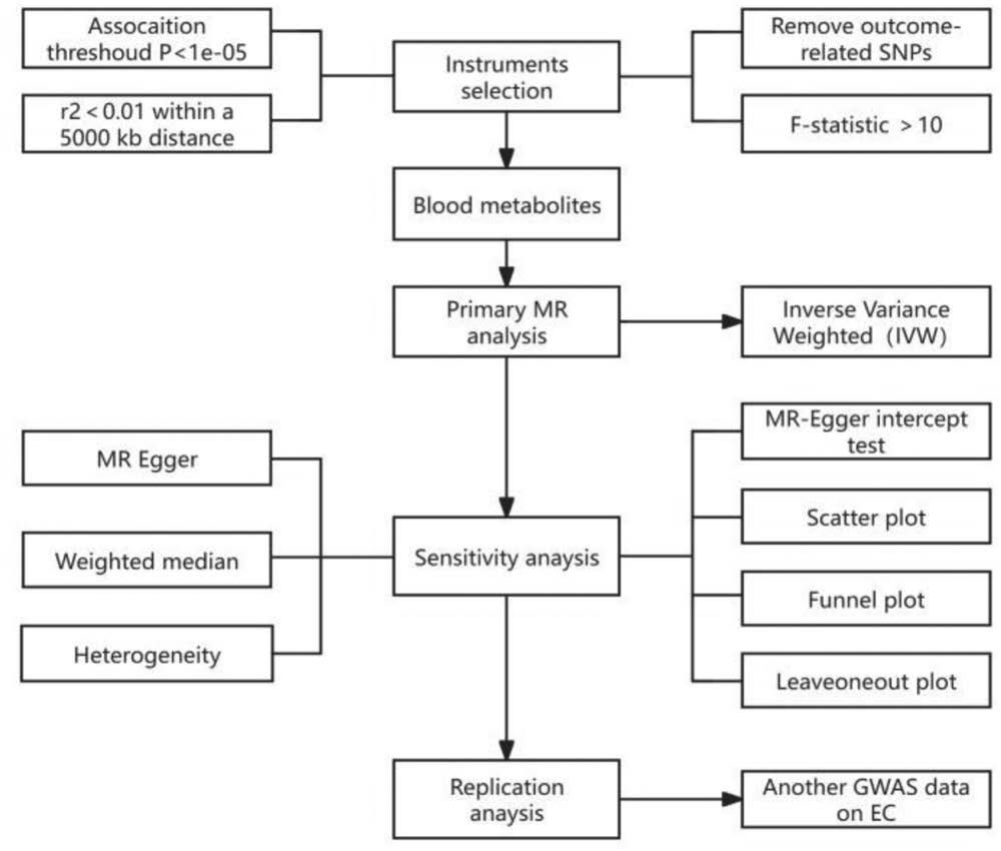
A flow chart showing the causal relationship between human blood metabolites and EC risk. MR = Mendelian randomization, EC = esophageal cancer, SNPs = single nucleotide polymorphisms.

MR analysis of 2 samples was used to systematically evaluate the possible causal relationship between human blood metabolites and EC. The research flow chart is shown in Figure 1. A strong correlation between instrumental variables (IVs) and exposure is desirable, as are zero correlations with any potential confounders, and no correlations between IVs and outcomes other than exposure.

### 2.2. Genome-wide association study (GWAS) data for 1400 blood metabolites

The influence of metabolic processes on disease risk and potential therapeutic targets can be observed. In this study, GWAS data for human blood metabolites were obtained from Chen et al,^[18]^ who conducted a GWAS on 1091 metabolites and 309 metabolite ratios from a cohort of 8299 individuals in the Canadian Longitudinal Study of Aging. Of the 1091 plasma metabolites tested, 850 had known properties in 8 super-pathways (i.e., lipids, amino acids, xenobiotics, nucleotides, cofactors and vitamins, carbohydrates, peptides, and energy), with the remaining 241 categorized as unknown or “partially” characterized molecules. Of the 309 metabolite ratios tested, 143 metabolite ratios were identified in 69 loci.

### 2.3. GWAS data for esophageal cancer

The GWAS summary statistics for EC were obtained from a study conducted by Jiang L et al^[19]^ The Fast GWA-GLMM method was utilized to analyze the UKB data, which consisted of 456,348 individuals of European ancestry. The GWAS catalog number for this study is GCST90041891.

To further validate the study results, another GWAS data from FINNGEN was repeated with MR (https://storage.googleapis.com/finngen-public-data-r10/summary_stats/finngen_R10_C3_OESOPHAGUS_EXALLC.gz).

### 2.4. Selection of IVs

In order to identify IVs associated with blood metabolites, stringent screening conditions were applied. Initially, the association threshold commonly^[20]^ used in previous MR studies was relaxed due to the limited number of single nucleotide polymorphisms (SNPs) with significant genome-wide effects. We used a relaxed association threshold (*P* < 1 × 10^‐5^) to ensure that sufficient SNPs could be obtained as IVs with limited sample size. The choice of relaxed thresholds was based on common practice in previous studies. To cope with possible bias due to weaker IVs, we used several methods in the sensitivity analysis, including MR-Egger regression and weighted median method, to verify the robustness of the results. Linkage disequilibrium (LD) analyses were conducted using the European (EUR) 1000 Genomes Project phase 3 reference panel to identify independent IVs based on a physical distance within 5000 kb and a linkage disequilibrium parameter of *r*^2^ < 0.01. Additionally, in order to mitigate potential bias arising from the use of weak IVs, the F-statistic was computed for each metabolite to assess the effectiveness of the IVs. To ensure an adequate level of variation for the metabolite of interest, IVs with an F-statistic below 10 were excluded. Furthermore, SNPs linked to the exposure were extracted from the outcome data, and SNPs that exhibited a significant association with the outcome (*P* < 1 × 10^‐5^) were excluded. The reconciliation of SNP exposure and SNP outcome alleles involved the removal of palindromic SNPs with moderately effective allele frequencies, as well as those containing incompatible alleles. Lastly, a MR analysis was conducted on metabolites that had more than 2 SNPs available for analysis in the MR framework.

### 2.5. MR analysis

The study employed a random-effects inverse variance weighting (IVW)^[21,22]^ approach to examine the causal relationship between blood metabolites and EC. The IVW method was chosen as it assumes no horizontal pleiotropy across all SNPs, thus providing the most accurate assessment of causal effects. The rationale for choosing the random-effects IVW model is its ability to provide more robust estimates in the presence of heterogeneity, and the random-effects model allows effect sizes to vary across studies compared to the fixed effects IVW model, thus better accommodating the real-world situation. According to IVW, exposure and outcome causal effects can be consistently estimated when the genetic variants satisfy the 3 IV assumptions and are not influenced by pleiotropy. The *P* value for the multiple hypothesis test corrected for Bonferroni was *P* < 1.03 × 10^‐4^ (0.05/1400), indicating a significant causal relationship. Moreover, *P* < .05 was considered nominally significant, and the selected metabolites could provide an indication of the risk of EC.

### 2.6. Sensitivity analysis

As a supplement to the MR methods, the study used MR-Egger,^[23,24]^ weighted median and MR-PRESSO in order to determine whether potential pleiotropy existed. These methods evaluated causal relationships using different postulated models. Additionally, the heterogeneity was evaluated using Cochran *Q* value. A *P* value of <.05 indicated the presence of heterogeneity. Furthermore, the MR-Egger intercept was computed to examine directional pleiotropy and bias resulting from invalid IVs.^[23]^ A *P* value <.05 suggested that horizontal pleiotropy influenced the IVW method, thereby questioning the reliability of the study findings. Subsequently, we performed a leave-one-out analysis (leave-one-out analysis) to assess whether any specific SNPs were unduly influencing the results. By removing each SNP in turn and rerunning the analysis, we were able to see if any 1 SNP significantly altered the results. The results showed that no single SNP significantly affected the final results, indicating the robustness of our findings. Sensitivity assessment was further supported by the inclusion of funnel plots and scatter plots.

### 2.7. Replication analysis

In order to comprehensively evaluate the reliability of the candidate metabolites identified according to the aforementioned criteria, we conducted a replication of the IVW analysis using a separate GWAS dataset from FINNGEN. The meta-analysis of the 2 MR analyses ultimately confirmed the identification of blood metabolites that are causally linked to EC. The meta-analysis was conducted using the random-effects IVW model, employing Review Manager 5.4 software.

## 3. Results

### 3.1. IVs’ strength

Through a GWAS of metabolite levels, we discovered 1509 associations involving 647 metabolites that met a rigorous Bonferroni correction threshold. Among the 1400 blood metabolites examined, we identified 75 IVs that exhibited strong associations with EC, with IVs SNP numbers ranging from 15 to 37. Notably, all metabolite-related SNPs possessed F-statistics exceeding 10, indicating a robust correlation between the IVs and EC.

### 3.2. Effects of genetically determined metabolites on EC

Initially, the IVW method (*P* < .05) was employed to analyze a total of 75 EC-related metabolites. These metabolites encompassed 6 distinct categories, namely 14 metabolite ratios, 33 lipids (including 11 fatty acyls, 6 steroids, 4 sphingolipids, 11 glycerophospholipids, and 1 glycerolipids), 4 organoheterocyclic compounds, 12 organic acids (comprising 7 amino acids, 2 organic sulfuric acids, 2 hydroxy acids, and 1 dipeptide), 1 nucleotide, 1 phenylpropanoids, 1 carbohydrates, and 9 unknown metabolites (Table 1). The results after Bonferroni correction were in line with the adjustment threshold and had statistical significance.

**Table 1:** Supplementary and sensitivity analyses of blood metabolites for EC causality.

Metabolites	Nsnp	MR analysis		MR-Egger		Weighted median		MR-Egger intercept	Heterogeneity
		OR (95% CI)	*P*	OR (95% CI)	*P*	OR (95% CI)	*P*	*P*	*P*
***Organic acids***									
**Amino acid**									
Beta-citrylglutamate	26	1.455 (1.015–2.085)	.041	1.526 (0.826–2.818)	.19	1.492 (0.942–2.364)	.088	.747	.853
N-acetyl arginine	26	0.734 (0.554–0.973)	.031	0.876 (0.573–1.339)	.547	0.798 (0.550–1.157)	.234	.287	.717
Hexanoylglycine	25	1.596 (1.076–2.366)	.020	0.613 (0.276–1.360)	.241	1.748 (1.064–2.872)	.027	.015	.214
3-Hydroxyphenylacetoylglutamine	17	2.000 (1.113–3.592)	.020	1.007 (0.309–3.282)	.992	2.234 (1.010–4.941)	.047	.210	.772
N-Acetyl-2-aminoadipate	24	0.645 (0.430–0.968)	.034	0.628 (0.314–1.256)	.202	0.718 (0.414–1.247)	.24	.927	.584
Dimethylarginine (sdma + adma)	20	1.869 (1.012–3.452)	.046	3.294 (0.868–12.497)	.097	1.490 (0.618–3.589)	.374	.360	.347
3-Methyl-2-oxobutyrate	21	0.477 (0.267–0.853)	.012	0.250 (0.049–1.291)	.114	0.517 (0.226–1.179)	.117	.420	.527
**Organic sulfuric acids**									
Dihydroferulic acid sulfate	26	0.579 (0.367–0.912)	.019	0.666 (0.249–1.783)	.427	0.564 (0.298–1.067)	.078	.754	.273
Tyramine O-sulfate	22	1.867 (1.063–3.280)	.030	1.955 (0.508–7.515)	.341	1.763 (0.819–3.796)	.147	.942	.686
**Hydroxy acids**									
2-Hydroxysebacate	20	0.568 (0.332–0.971)	.039	0.840 (0.249–2.837)	.781	0.654 (0.298–1.436)	.29	.758	.492
2-Hydroxyglutarate	27	0.512 (0.299–0.878)	.015	0.221 (0.053–0.917)	.048	0.556 (0.268–1.153)	.115	.223	.265
**Dipeptide**									
Gamma-glutamylmethionine	25	0.461 (0.261–0.815)	.008	0.206 (0.044–0.963)	.056	0.368 (0.164–0.826)	.015	.283	.436
***Lipid***									
**Fatty acyls**									
Phytanate	21	0.463 (0.251–0.855)	.014	0.474 (0.130–1.731)	.273	0.467 (0.212–1.030)	.059	.968	1.029
Eicosapentaenoate (EPA; 20:5n3)	26	2.113 (1.274–3.505)	.004	1.561 (0.549–4.437)	.411	3.420 (1.635–7.152)	.001	.522	.569
Hydroxy-cmpf	19	0.417 (0.209–0.833)	.013	0.204 (0.0348–1.199)	.097	0.504 (0.209–1.219)	.128	.401	.221
Docosatrienoate (22:3n3)	22	0.620 (0.390–0.986)	.044	0.278 (0.104–0.741)	.019	0.671 (0.342–1.316)	.246	.084	.862
Dihomo-linoleoylcarnitine (C20:2)	29	1.578 (1.001–2.487)	.049	1.510 (0.592–3.856)	.396	1.359 (0.726–2.542)	.337	.917	.165
Malonylcarnitine	21	2.152 (1.266–3.656)	.005	3.066 (0.792–11.872)	.121	1.860 (0.879–3.933)	.104	.584	.589
Octadecenedioate (C18:1-DC)	24	0.620 (0.427–0.905)	.012	0.372 (0.194–0.716)	.007	0.608 (0.369–1.001)	.050	.076	.746
Octadecadienedioate (C18:2-DC)	34	0.664 (0.487–0.906)	.010	0.687 (0.417–1.134)	.152	0.652 (0.416–1.024)	.063	.866	.807
Octadecenedioylcarnitine (C18:1-DC)	18	0.627 (0.400–0.983)	.042	0.808 (0.397–1.644)	.564	0.623 (0.398–0.975)	.038	.325	.094
Octadecanedioylcarnitine (C18-DC)	28	0.594 (0.430–0.820)	.002	0.648 (0.372–1.130)	.138	0.629 (0.392–1.009)	.055	.706	.884
Carnitine C5:1	26	1.828 (1.083–3.085)	.024	1.342 (0.364–4.944)	.663	1.775 (0.872–3.612)	.113	.616	.956
**Steroids**									
Glycochenodeoxycholate glucuronide (1) l	25	0.761 (0.591–0.981)	.035	0.800 (0.559–1.145)	.234	0.827 (0.591–1.156)	.266	.706	.721
Glycolithocholate	23	1.814 (1.051–3.131)	.033	2.840 (0.866–9.304)	.100	1.761 (0.822–3.773)	.146	.414	.549
Glycocholenate sulfate	37	0.754 (0.569–0.999)	.049	0.708 (0.450–1.112)	.143	0.677 (0.457–1.002)	.051	.726	.418
5alpha-androstan-3alpha,17alpha-diol monosulfate	28	1.488 (1.062–2.085)	.021	1.5267 (0.832–2.802)	.184	1.759 (1.067–2.899)	.027	.922	.684
Androstenediol (3beta,17beta) monosulfate (2)	27	1.889 (1.133–3.148)	.015	0.557 (0.173–1.797)	.337	2.287 (1.098–4.763)	.027	.032	.537
Deoxycholic acid 12-sulfate	22	0.631 (0.413–0.965)	.034	0.709 (0.317–1.588)	.413	0.575 (0.317–1.043)	.069	.740	.275
**Sphingolipids**									
Sphingomyelin (d18:2/24:1, d18:1/24:2)	34	1.988 (1.184–3.339)	.009	1.597 (0.452–5.644)	.473	2.449 (1.200–5.001)	.014	.712	.936
Sphingomyelin (d18:2/14:0, d18:1/14:1)	22	2.753 (1.366–5.551)	.005	2.015 (0.453–8.968)	.369	1.894 (0.725–4.952)	.193	.647	.972
Glycosyl-N-(2-hydroxynervonoyl)-sphingosine (d18:1/24:1 (2OH))	25	1.536 (1.000–2.360)	.0498	1.340 (0.545–3.296)	.53	1.317 (0.703–2.468)	.389	.738	.517
Sphingomyelin (d18:2/23:0, d18:1/23:1, d17:1/24:1)	25	2.490 (1.350–4.594)	.004	1.625 (0.375–7.038)	.523	3.522 (1.434–8.650)	.006	.535	.456
**Glycerophospholipids**									
1-Palmitoyl-GPC (16:0)	26	0.564 (0.322–0.988)	.045	0.359 (0.090–1.438)	.160	0.678 (0.318–1.445)	.314	.492	.212
1-Lignoceroyl-GPC (24:0)	15	2.010 (1.028–3.935)	.041	0.621 (0.104–3.719)	.611	2.479 (0.975–6.295)	.056	.188	.903
1-Palmitoleoyl-2-linolenoyl-GPC (16:1/18:3)	18	0.535 (0.321–0.893)	.017	0.647 (0.208–2.013)	.463	0.410 (0.197–0.852)	.017	.716	.418
1-Linolenoyl-GPC (18:3)	30	0.489 (0.298–0.802)	.005	1.048 (0.337–3.259)	.937	0.538 (0.263–1.101)	.090	.154	.806
1,2-Dilinoleoyl-GPC (18:2/18:2)	15	0.457 (0.273–0.765)	.003	0.363 (0.145–0.904)	.049	0.444 (0.229–0.862)	.016	.558	.992
1-Oleoyl-GPG (18:1)	24	0.618 (0.424–0.901)	.012	0.486 (0.239–0.990)	.059	0.595 (0.344–1.027)	.062	.444	.780
1-Linoleoyl-GPG (18:2)	24	0.567 (0.367–0.876)	.011	0.398 (0.192–0.827)	.022	0.581 (0.323–1.046)	.070	.254	.237
1-Palmitoyl-2-arachidonoyl-GPE (16:0/20:4)	19	1.493 (1.078–2.067)	.016	1.256 (0.702–2.247)	.452	1.330 (0.864–2.046)	.195	.492	.788
1-Linoleoyl-GPE (18:2)	32	0.518 (0.346–0.775)	.001	0.393 (0.172–0.895)	.034	0.407 (0.217–0.762)	.005	.456	.931
1-Stearoyl-2-arachidonoyl-GPE (18:0/20:4)	30	1.667 (1.222–2.272)	.001	1.540 (0.874–2.714)	.146	1.534 (0.993–2.368)	.054	.748	.511
1-Stearoyl-2-arachidonoyl-GPI (18:0/20:4)	24	1.709 (1.100–2.654)	.017	1.646 (0.609–0.608)	.337	2.072 (1.124–3.816)	.019	.934	.275
**Glycerolipids**									
1-Arachidonylglycerol (20:4)	22	2.009 (1.242–3.251)	.004	1.612 (0.619–4.199)	.340	2.089 (1.049–4.160)	.036	.607	.951
***Phenylpropanoids***									
3-(3-Hydroxyphenyl)propionate	20	0.504 (0.277–0.916)	.024	0.545 (0.143–2.076)	.385	0.456 (0.206–1.011)	.053	.898	.843
***Organoheterocyclic compounds***									
Sulfate of piperine metabolite C_16_H_19_NO_3_ (3)	27	1.743 (1.034–2.937)	.037	1.43 (0.381–5.366)	.601	1.402 (0.676–2.908)	.364	.752	.561
5-Hydroxy-2-methylpyridine sulfate	19	1.939 (1.098–3.424)	.022	1.648 (0.436–6.225)	.471	1.832 (0.823–4.080)	.138	.794	.651
1-Methyl-4-imidazoleacetate	28	1.558 (1.006–2.411)	.047	0.831 (0.222–3.108)	.787	0.722 (0.359–1.452)	.361	.539	.129
Metabolonic lactone sulfate	31	0.707 (0.530–0.942)	.018	0.641 (0.421–0.977)	.048	0.648 (0.440–0.953)	.028	.539	.295
***Nucleotide***									
5-Methyluridine (ribothymidine)	25	1.458 (1.041–2.041)	.028	1.200 (0.726–1.983)	.485	1.305 (0.859–1.982)	.212	.316	.885
***Carbohydrate***									
Sucrose	17	0.441 (0.224–0.867)	.018	0.962 (0.217–4.264)	.960	0.431 (0.165–1.124)	.085	.266	.699
***Metabolite ratio***									
Adenosine 5’-diphosphate (ADP) to 5-oxoproline	17	0.568 (0.323–0.998)	.0492	0.831 (0.222–3.108)	.787	0.722 (0.363–1.436)	.353	.539	.129
Adenosine 5’-diphosphate (ADP) to mannose	18	0.542 (0.333–0.882)	.014	0.652 (0.199–2.134)	.489	0.692 (0.351–1.365)	.288	.742	.531
2’-Deoxyuridine to cytidine	26	1.686 (1.013–2.805)	.044	0.848 (0.294–2.449)	.764	1.882 (0.908–3.898)	.089	.161	.87
Glutamate to kynurenine	26	1.789 (1.033–3.097)	.038	0.748 (0.211–2.659)	.658	1.922 (0.883–4.184)	.100	.148	.616
Phosphate to tryptophan	20	2.036 (1.064–3.896)	.031	3.724 (0.614–22.565)	.170	2.032 (0.827–4.994)	.122	.491	.746
Adenosine 5’-monophosphate (AMP) to tyrosine	18	0.447 (0.23–0.869)	.018	0.455 (0.093–2.226)	.345	0.459 (0.180–1.170)	.103	.981	.455
Adenosine 5’-monophosphate (AMP) to EDTA	15	0.437 (0.203–0.942)	.035	0.255 (0.046–1.415)	.142	0.379 (0.137–1.051)	.062	.410	.5
Phosphate to N-palmitoyl-sphingosine (d18:1 to 16:0)	23	0.564 (0.318–0.999)	.0497	0.733 (0.181–2.974)	.669	0.762 (0.344–1.687)	.502	.691	.826
Benzoate to linoleoyl-arachidonoyl-glycerol (18:2 to 20:4) [1]	16	0.594 (0.366–0.962)	.034	0.368 (0.122–1.108)	.097	0.467 (0.242–0.902)	.023	.360	.598
Cholesterol to linoleoyl-arachidonoyl-glycerol (18:2 to 20:4) [2]	30	0.595 (0.388–0.912)	.017	0.502 (0.209–1.207)	.135	0.436 (0.230–0.826)	.011	.668	.275
Arachidonate (20:4n6) to linoleate (18:2n6)	24	1.696 (1.156–2.489)	.007	1.628 (0.870–3.045)	.142	1.841 (1.156–2.929)	.01	.872	.508
Retinol (Vitamin A) to linoleoyl-arachidonoyl-glycerol (18:2 to 20:4) [1]	24	0.487 (0.317–0.750)	.001	0.871 (0.371–2.047)	.755	0.482 (0.257–0.903)	.023	.140	.385
Cholesterol to cortisol	15	0.443 (0.214–0.916)	.028	0.368 (0.122–1.108)	.097	0.467 (0.242–0.902)	.023	.360	.598
3-Hydroxyisobutyrate to phosphate	24	0.376 (0.210–0.671)	.001	0.302 (0.086–1.067)	.076	0.329 (0.148–0.732)	.006	.708	.667
***Unknown***									
X-25810	35	0.621 (0.392–0.983)	.042	0.621 (0.253–1.520)	.304	0.604 (0.314–1.160)	.13	.997	.529
X-24418	24	1.451 (1.015–2.072)	.041	1.212 (0.655–2.245)	.546	1.065 (0.64–1.772)	.809	.492	.986
X-24757	21	1.872 (1.036–3.38)	.038	1.081 (0.234–4.987)	.922	1.991 (0.925–4.286)	.078	.454	.167
X-23639	22	1.839 (1.056–3.202)	.031	1.497 (0.366–6.129)	.581	1.594 (0.751–3.385)	.225	.759	.749
X-26111	22	0.521 (0.289–0.939)	.030	0.841 (0.256–2.76)	.778	0.512 (0.235–1.118)	.093	.373	.052
X-21467	36	0.65 (0.45–0.94)	.022	0.492 (0.264–0.92)	.033	0.702 (0.429–1.148)	.158	.289	.121
X-23678	20	0.540 (0.320–0.911)	.021	0.358 (0.122–1.053)	.078	0.612 (0.284–1.32)	.211	.561	.405
X-23641	30	0.608 (0.403–0.918)	.0181	0.438 (0.141–1.358)	.164	0.802 (0.445–1.447)	.464	.546	.388
X-12701	15	0.355 (0.191–0.661)	.001	0.335 (0.079–1.417)	.161	0.276 (0.113–0.672)	.005	.931	.736

**Figure 2.**
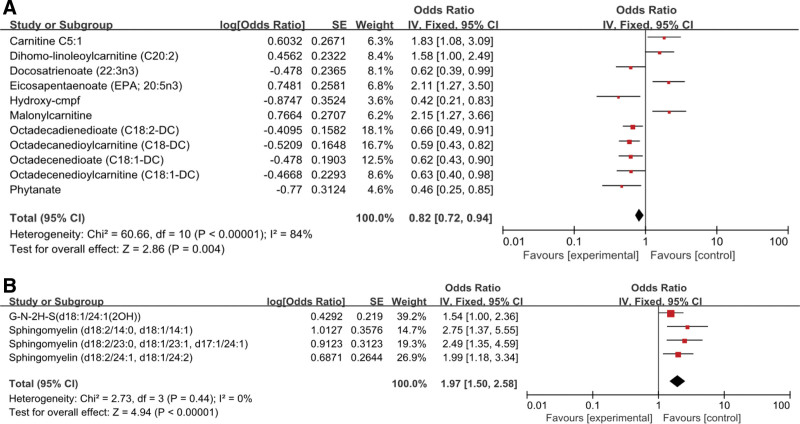
(A and B) Forest plot for the causal effect of blood metabolites on the risk of EC. OR = odds ratio, CI = confidence interval. A: fatty acyls; B: sphingolipids.

Then, we assessed heterogeneity and pleiotropy using a variety of sensitivity analyses. May reduce the risk of EC, including 1 carbohydrate (odds ratio [OR] = 0.44, 95% CI = 0.22–0.87, *P* < .02), 1 dipeptide (OR = 0.46, 95% confidence interval [CI] = 0.26–0.81, *P* < .008), 7 fatty acyls (OR = 0.82, 95% CI = 0.72–0.94, *P* < .004), 2 hydroxyl acids (OR = 0.54, 95% CI = 0.37–0.79, *P* < .001), 10 metabolite ratios (OR = 0.77, 95% CI = 0.67–0.8, *P* < .0002), and 6 unknown metabolites (OR = 0.81, 95% CI = 0.7–0.95, *P* < .009). May increase the risk of EC. One nucleoside (OR = 1.46, 95% CI = 1.04–2.04, *P* < .03) and 4 sphingolipids (OR = 1.97, 95% CI = 1.5–2,85, *P* < .00001) were included, and their *P* values were statistically significant (Fig. 2).

### 3.3. Replication analysis

**Figure 3.**
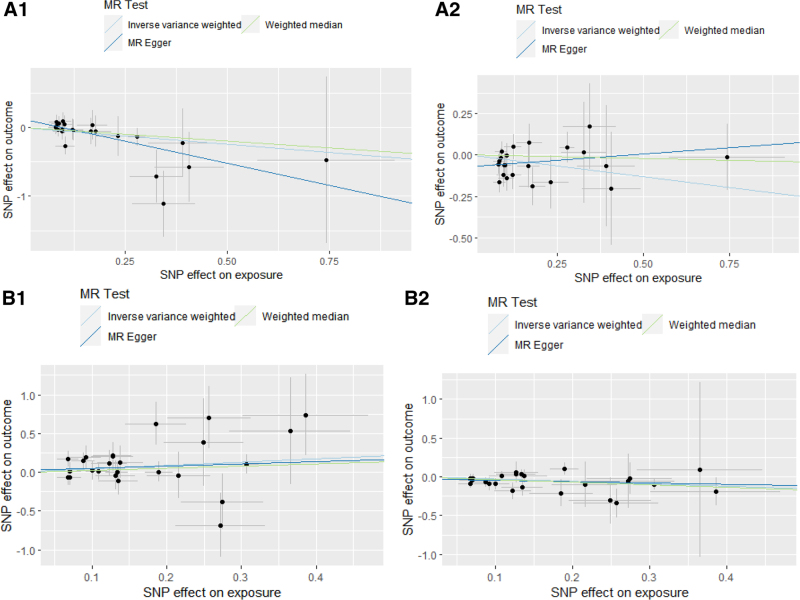
(A1/A2/B1/B2) MR scatter-plot heterogeneity analysis of overlapped metabolite on esophageal cancer. (A1) Docosatrienoate (22:3n3) on EC (from Nielsen et al); (A2) Docosatrienoate (22:3n3) on EC (from FINNGEN); (B1) glycosyl-N-(2-hydroxynervonoyl)-sphingosine (d18:1/24:1 (2OH)) on EC (from Nielsen et al); (B2) glycosyl-N-(2-hydroxynervonoyl)-sphingosine (d18:1/24:1 (2OH)) on EC (from FINNGEN).

**Figure 4.**
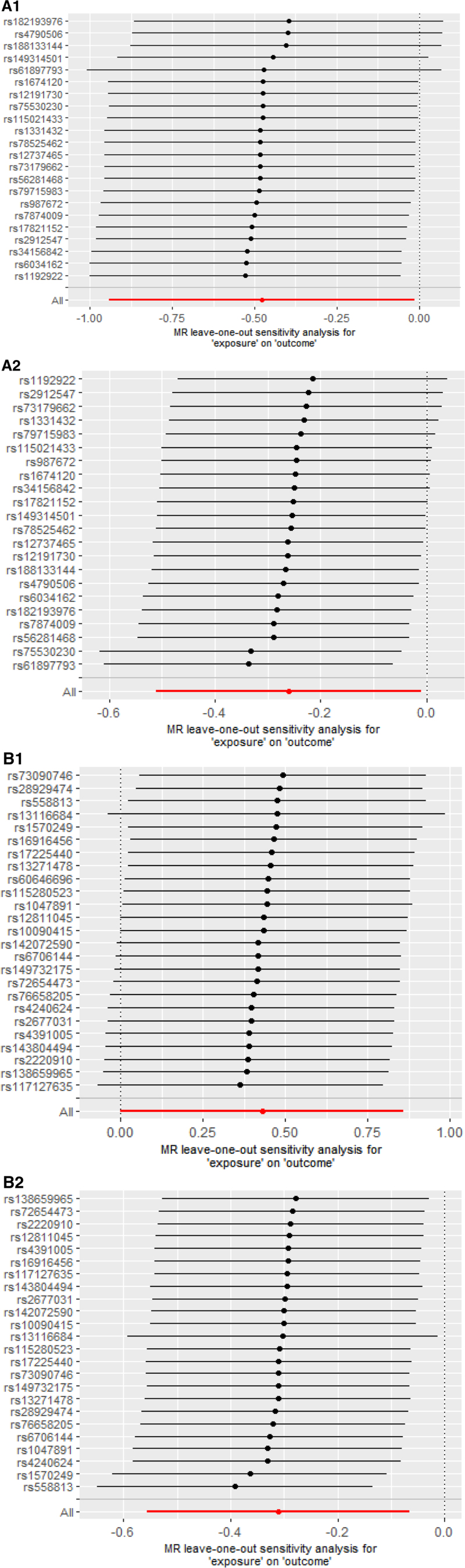
(A1/A2/B1/B2) Forest plots for leave-one-out analysis of overlapped metabolite o on esophageal cancer. (A1) Docosatrienoate (22:3n3) on EC (from Nielsen et al); (A2) Docosatrienoate (22:3n3) on EC (from FINNGEN); (B1) glycosyl-N-(2-hydroxynervonoyl)-sphingosine (d18:1/24:1 (2OH)) on EC (from Nielsen et al); (B2) glycosyl-N-(2-hydroxynervonoyl)-sphingosine (d18:1/24:1 (2OH)) on EC (from FINNGEN).

To further validate the results, another set of GWAS data from the EC in Finland was repeated using the same method. Firstly, a total of 43 blood metabolites were screened by IVW method, including 9 lipids, 3 compounds, 12 organic acids, 10 blood metabolite ratios, and 9 unknown metabolites. There were 2 replicates in the 2 groups: Docosatrienoate (22:3n3) and glycosyl-N-(2-hydroxynervonoyl)-sphingosine (d18:1/24:1 (2OH)), both of which were lipids. Docosatrienoate (22:3n3) was causally associated with reduced risk of ES, the EC GWAS data (from Jiang et al) (OR = 0.620, 95% CI = 0.390–0.986, *P* = .044) and the EC GWAS data (from FINNGEN) (OR = 0.77, 95% CI = 0.6–0.99, *P* = .042). But compare the results between the 2 groups. Glycosyl-N-(2-hydroxynervonoyl)-sphingosine (d18:1/24:1 (2OH)) shows the opposite result, the EC GWAS data (from Jiang L et al) (OR = 1.536,95% CI = 1.000–2.360, *P* = .049), While the EC GWAS data (from FINNGEN) (OR = 0.733, 95% CI = 0.574–0.937, *P* = .013) (Table 2). The results obtained from the IVW method demonstrate statistical significance (*P* < .05), with consistent findings in terms of direction and magnitude across IVW, MR-Egger, and weighted median approaches (Fig. 3). Furthermore, the Cochran *Q* test (*P* > .05) and MR-Egger intercept test (*P* > .05) indicated the absence of statistically significant heterogeneity and pleiotropy (Fig. 4).

**Table 2:** Metabolites that overlap in esophageal cancer sensitivity analysis.

Metabolite	MR analysis		MR-Egger		Weighted median		MR-Egger intercept
	OR (95%Cl)	*P*	OR (95%Cl)	*P*	OR (95%Cl)	*P*	*P*
**Docosatrienoate (22:3n3**)							
Jiang L et al	0.620 (0.390–0.986)	.044	0.278 (0.104–0.741)	.019	0.671 (0.342–1.316)	.246	.084
FINNGEN	0.77 (0.6–0.99)	.042	1.63 (0.737–1.835)	.524	0.957 (0.653–1.403)	.823	.047
**Glycosyl-N-(2-hydroxynervonoyl)-sphingosine (d18:1/24:1 (2OH**))							
Jiang L et al	1.536 (1.000–2.360)	.049	1.340 (0.545–3.296)	.53	1.317 (0.703–2.468)	.389	.738
FINNGEN	0.733 (0.574–0.937)	.013	0.842 (0.51–1.39)	.508	0.719 (0.487–1.063)	.098	.541

## 4. Discussion

In this study, we used large-scale GWAS data from public databases to investigate the causal relationship between 1400 blood metabolites and the risk of esophageal neoplasia using unbiased two-sample MR analyses. However, after rigorous quality control, the results confirmed a direct causal association between certain circulating substances in this blood and the development of EC. The results showed 2 overlapping potential predictors of EC risk, and both were lipids. These included 1 metabolite fatty acyl (Docosatrienoate (22:3n3)) that may be associated with a reduced risk of esophageal tumorigenesis, which reached statistical significance in both 2 MR models with consistent findings. However, the research results for another metabolite, glycosyl-N-(2-hydroxynervonoyl)-sphingosine (d18:1/24:1 (2OH)), are inconsistent between the 2 datasets.

Esophageal squamous cell carcinoma (ESCC) is relatively common in China and is characterized by high malignancy and poor prognosis.^[25,26]^ Studying the correlation between ESCC and associated metabolites within the body can aid in the advancement of strategies for the prevention, early detection, and treatment of ESCC. The aberrant lipid metabolism observed during tumorigenesis and progression has become a subject of growing interest in recent years. Key enzymes involved in lipid uptake, de novo synthesis, and catabolism exhibit aberrant overexpression or overactivation in various cancer types, facilitating tumor cell proliferation, invasion, and metastasis.^[27]^ Given the crucial role of lipids as a primary energy source and vital cellular components, it is evident that cancer progression, characterized by uncontrolled cell growth and proliferation, is intricately associated with lipid metabolism.^[28,29]^ The results of the 2-group MR study in this study showed a causal relationship between lipids and EC risk.

Fatty acyl groups are structurally the simplest lipids and contain a wealth of information on fatty acid metabolism covering lipid uptake, biosynthesis, lipolysis, and lipid oxidation. In this study, we found that docosatrienoic acid (Docosatrienoate (22:3n3)) may act as a potential protective substance, thereby reducing the risk of EC development. Docosatrienoic acid belongs to the omega-3 family of polyunsaturated fatty acids, which are mainly found in some fish, seaweeds and vegetable oils. In recent years, intervention strategies through supplementation with specific nutrients, rather than dietary restriction, have also become a new direction in the exploration of tumor therapy. Study finds that adding ω-3 polyunsaturated fatty acids (PUFA) to the diet of mice also exerts a potential anti-tumor effect.^[30]^ Meanwhile, Dierge, E et al^[31]^ found that if dietary PUFA intake is increased, it will promote lipid peroxidation of cancer cells in the acidic tumor microenvironment and induce iron death of cancer cells, thus exerting anti-tumor effects. A randomized, double-blind trial by Ryan, AM et al^[32]^ also demonstrated better preservation of lean tissue in the omega-3 PUFA enteral nutrition group compared to the standard enteral nutrition group in perioperative EC patients, and Moses et al^[33]^ demonstrated that pancreatic cancer patients who received omega-3 PUFA for 8 weeks showed significant improvements in total energy expenditure and physical activity levels compared to pancreatic cancer patients who did not receive omega-3 PUFA supplementation. This indicates that docosahexaenoic acid may play a significant role in mitigating the likelihood of EC.

Glycosyl-N-(2-hydroxyneuramoyl)-sphingosine belongs to the sphingolipid class, and emerging evidence suggests that sphingolipids play a role in physiological processes and cancer development.^[34,35]^ It has been proposed that sphingolipids can modulate signaling functions within cancer cell signal transduction networks, thereby regulating processes such as growth, proliferation, migration, invasion, and metastasis.^[36,37]^ Sphingosine is phosphorylated by sphingosine kinase, also known as SK1 (SPHK1) or SPHK2 (also known as SK2) to produce S1P, which binds to the 5 specific G-protein-coupled receptors (gpcr) s1pr 1-5 (also known as S1 P1-5) in an autocrine or paracrine manner in a variety of cancer cells Elicits pro-survival signaling.^[38–40]^ Studies have demonstrated that increased expression of SPHK1 can promote tumor migration, invasion, and angiogenesis via various pathways, including the SPHK1/miR-144-3p/FN1 and SPHK1/p-PAK axes.^[41,42]^ Upregulation of SPHK1 has been documented in several malignancies, such as gastric cancer,^[43]^ breast cancer,^[44]^ lung cancer,^[45]^ brain tumors,^[46]^ colon cancer,^[47]^ and lymphomas.^[48]^ Moreover, previous studies have demonstrated a correlation between SPHK1 expression and human tumor progression and unfavorable clinical outcomes.^[49–51]^ Nigel J Pyne and colleagues provided evidence indicating that elevated levels of SPHK1 protein were correlated with a less favorable prognosis.^[52]^ SubbaRao et al demonstrated that inhibition of SPHK1 resulted in decreased phosphorylated Akt levels and cell cycle arrest in the G0/G1 phase in melanoma cells, ultimately leading to decreased tumor cell proliferation and apoptosis.^[53]^ This is consistent with our analysis showing an increased expression of sphingolipids in EC tissue (Table 1), where glycosyl-N-(2-hydroxyceramide)-sphingosine may increase the risk of EC. Interestingly, in another set of experimental data from this study (Table 2), we observed that glycosyl-N-(2-hydroxyceramide)-sphingosine might have a potential protective effect in reducing the risk of esophageal tumors. The reasons for this discrepancy may include several points: first, since the 2 sets of data in this study come from different populations with distinct genetic backgrounds and lifestyles, this could affect the relationship between glycosyl-N-(2-hydroxyceramide)-sphingosine and EC. At the same time, there may be unmeasured or insufficiently controlled confounding factors that may affect the estimation of the relationship between glycosyl-N-(2-hydroxyceryl)-sphingosine and EC in the 2 group analyses. Second, glycosyl-N-(2-hydroxyceramide)-sphingosine may play different roles in various biological pathways and environments. Studies have indicated that glycosyl-N-(2-hydroxyceramide)-sphingosine has complex functions in immune modulation, potentially promoting inflammation (increasing tumor risk) in certain circumstances and exhibiting anti-inflammatory effects (reducing tumor risk) in others.^[54]^ Furthermore, the impact of glycosyl-N-(2-hydroxyceramide)-sphingosine on the risk of EC may also depend on the specific type and stage of the disease. For instance, glycosyl-N-(2-hydroxyceramide)-sphingosine might exert a protective role in the early stages of EC, while in advanced stages, it may not have a significant effect or its role may be reversed. Lastly, glycosyl-N-(2-hydroxyceramide)-sphingosine may influence the risk of EC through multiple biological mechanisms, which could show varying effect sizes and directions in different populations and environments. However, no studies have been found in the existing literature regarding the beneficial effects of glycosyl-N-(2-hydroxyceramide)-sphingosine on EC, indicating a gap in this area of research. Therefore, further scientific research is needed to fully understand the precise role of glycosyl-N-(2-hydroxyceramide)-sphingosine as a protective substance in reducing the risk of EC, to confirm its mechanisms and effects. Future studies should focus on exploring the mechanisms of action of glycosyl-N-(2-hydroxyceramide)-sphingosine in different biological environments, assessing its dose–response relationship with the risk of EC, and verifying its potential protective role in different populations. Through these studies, we will not only gain a deeper understanding of the role of glycosyl-N-(2-hydroxyceramide)-sphingosine in the occurrence and development of EC but may also provide new strategies and targets for the prevention and treatment of EC.

## 5. Strengths and limitations

This study is innovative in the following ways: (1) this research examines the potential causal link between blood metabolites and the onset of EC through a molecular mechanism perspective, which is supported by robust theoretical foundations and holds significant clinical research implications. (2) Furthermore, stringent quality control measures and analytical techniques are employed in this study, alongside the utilization of diverse models to evaluate the causal impact, resulting in dependable and consistent findings. (3) In contrast to prior MR investigations focusing on a singular exposure factor, the present study examines a multitude of blood metabolites, presenting a substantial analytical burden and challenge.

There are some limitations in this study: the genetic data for EC in GWAS have primarily been sourced from European populations, necessitating further investigation among diverse populations. The risk predictors identified in preliminary analyses are predominantly unfamiliar metabolites with ambiguous functional characteristics. Despite efforts to utilize GWAS data with increased sample sizes, additional research is essential to enhance the accuracy of assessing the genetic influence of metabolites.

## 6. Conclusion

In conclusion, a two-sample MR method was employed to investigate the potential causal association between 1400 blood metabolites and EC. Findings suggest that the lipid metabolite docosatrienoic acid (22:3n3) may exhibit a protective effect against EC, thereby potentially reducing the risk of developing this malignancy. There is a potential correlation between glycosyl-N-(2-hydroxyneuramoyl)-sphingosine (d18:1/24:1 (2OH)) and the susceptibility to EC; however, additional research is required to validate the precise connection. Our findings offer novel perspectives on the involvement of genetic-exposure interactions in the development of EC.

## Acknowledgments

Thanks to all the investigators and participants who made their original GWAS results public.

## Author contributions

**Conceptualization:** Silin Wu, Ke Yu.

**Data curation:** Jieyin Deng, Yi Deng.

**Investigation:** Jieyin Deng, Ye Huang.

**Methodology:** Jieyin Deng, Ye Huang.

**Project administration:** Jieyin Deng, Silin Wu, Ye Huang.

**Resources:** Jieyin Deng.

**Software:** Jieyin Deng.

**Supervision:** Silin Wu, Ke Yu.

**Validation:** Silin Wu, Ke Yu.

**Writing – original draft:** Jieyin Deng, Silin Wu.

**Writing – review & editing:** Ke Yu.
